# SIRT1‐mediated ERβ suppression in the endothelium contributes to vascular aging

**DOI:** 10.1111/acel.12515

**Published:** 2016-07-29

**Authors:** Danli Kong, Ying Zhan, Zhaoyu Liu, Ting Ding, Min Li, Haibing Yu, Laxi Zhang, Huawen Li, Aiyue Luo, Dongwei Zhang, Yifei Wang, Shixuan Wang, Zhefan Zhang, Hongyu Zhang, Xiaodong Huang, Paul Yao, Yuanling Ding, Zhengxiang Liu

**Affiliations:** ^1^ School of Public Health Guangdong Medical College Dongguan 523808 China; ^2^ Tongji Wenchang Hospital Huazhong University of Science and Technology Wenchang 571321 China; ^3^ Tongji Hospital Huazhong University of Science and Technology Wuhan 430030 China; ^4^ Inner Mongolia University for the Nationalities #1742 Huolinhe Str. Tongliao Inner Mongolia 028000 China; ^5^ Guangzhou Biomedical Research and Development Center Jinan University Guangzhou 510632 China; ^6^ Personalized Treatment Research Center The Third Hospital of Wuhan Wuhan 430060 China; ^7^ Department of Hematology Peking University ShenZhen Hospital ShenZhen 518036 China

**Keywords:** estrogen receptor, fatty acid metabolism, mitochondria, oxidative stress, vascular aging

## Abstract

SIRT1 has many important molecular functions in aging, and the estrogen receptors (ERs) have a vasculoprotective effect, although the detailed mechanism for the roles of SIRT1 and ERs in vascular aging remains unclear. We found that ERβ expression in the endothelium was reduced in aging mice, and the expression of ERα and SIRT1 did not change, while SIRT1 activity declined. Further investigation showed that the ERβ expression was regulated by SIRT1 through complexes of SIRT1‐PPARγ/RXR‐p300 that bind to a PPRE (PPAR response element) site on the ERβ promoter, and the declined SIRT1 function in aging mice was due to compromised phosphorylation at S154. A single‐mutant SIRT1‐C152(D) restored the reduced ERβ expression in the endothelium with minimized reactive oxygen species generation and DNA damage and increased mitochondrial function and fatty acid metabolism. In high‐fat diet aging mice, the endothelium‐specific delivery of ERβ or SIRT1‐C152(D) on the vascular wall reduced the circulating lipids with ameliorated vascular damage, including the restored vessel tension and blood pressure. We conclude that SIRT1‐mediated ERβ suppression in the endothelium contributes to vascular aging, and the modulation of SIRT1 phosphorylation through a single‐mutant SIRT1‐C152(D) restores this effect.

## Introduction

SIRT1 is an NAD‐dependent histone deacetylase. It has many important molecular functions and is considered an important protein in aging and metabolic regulations (Imai & Guarente, 2014). Vascular aging is a dominant risk factor in vascular diseases, and many structural and functional changes are involved in this process. This includes vascular stiffness, increased secretion of inflammatory molecules, accumulation of oxidative stress, endothelial dysfunction, hypertension, and atherosclerosis development (Sindler *et al*., 2011). SIRT1 plays an important role in vascular endothelial dysfunction in aging (Vinciguerra *et al*., 2012), although the detailed mechanism remains unclear.

The analysis of mouse models targeted at ERα or ERβ demonstrated a prominent role of ERα in vascular biology (Arnal *et al*., 2010). ERα plays a dominant role in E2‐induced vasculoprotective effect, while in using ERα and/or ERβ null mice, both ERs have been shown to be necessary and sufficient for E2‐mediated vascular protection (Couse & Korach, 1999). ERβ plays an essential role in the regulation of the vascular function and cardioprotective effect and contributes to the development of atherosclerosis and vascular aging, although the detailed mechanism still needs to be fully understood (Zhu *et al*., 2002; Lin *et al*., 2009). The above contradictory observations can be well explained by our recent findings. We have previously shown that the SOD2 (mitochondrial superoxide dismutase) expression is mediated through E2‐induced ERα/ERβ activation, where ERα is responsible for E2‐mediated SOD2 activation, and ERβ is responsible for E2‐mediated SOD2 basal expression in vascular endothelial cells. Knockdown of ERα does not significantly decrease the SOD2 basal expression (Liu *et al*., 2014). Furthermore, our recent work showed the similar mechanism for E2‐induced ERRα (estrogen‐related receptor α) expression (Li *et al*., 2015). This indicates that ERα and ERβ both play important roles in E2‐induced vasculoprotective action.

ERRα is an orphan nuclear receptor that shares some identical sequence and the same target genes with estrogen receptor (ER). ERRα modulates the mitochondrial function by regulation of genes involved in mitogenesis, oxidative phosphorylation (Schreiber *et al*., 2004), and mitochondrial replication (Kelly & Scarpulla, 2004). It modulates the fatty acid metabolism by regulation of genes involved in fatty acid transportation/uptake and fatty acid β‐oxidation (Nakajima *et al*., 2013). It also modulates the vessel tension by regulation of eNOS expression (Sumi & Ignarro, 2003).

We found that ERβ expression in the endothelium from aging mice was reduced, and that there was no change in expression for ERα and SIRT1, while SIRT1 activity was declined, and further investigation showed that ERβ was regulated by SIRT1. Therefore, our study has the below hypothesis to connect SIRT1 and ERβ with the vascular function endpoints: In aging mice, the declined SIRT1 activity suppresses the ERβ expression and its target genes, including ERRα, SOD2, and eNOS, where ERRα expression regulates the mitochondrial function and fatty acid metabolism, SOD2 regulates the oxidative stress, and eNOS regulates the vessel tension. Subsequently, reduced ERRα expression contributes to the mitochondrial dysfunction and disorder of fatty acid metabolism; reduced SOD2 expression contributes to the oxidative stress and DNA break; and reduced eNOS expression contributes to the disorder of vessel tension. All of these consequences contribute significantly to vascular damage. Therefore, SIRT1‐mediated ERβ suppression in the endothelium contributes to vascular aging.

## Results

### ERβ expression in the vascular endothelium reduces in aging mice, while SIRT1 activity declines with no change in expression

**Figure 1:**
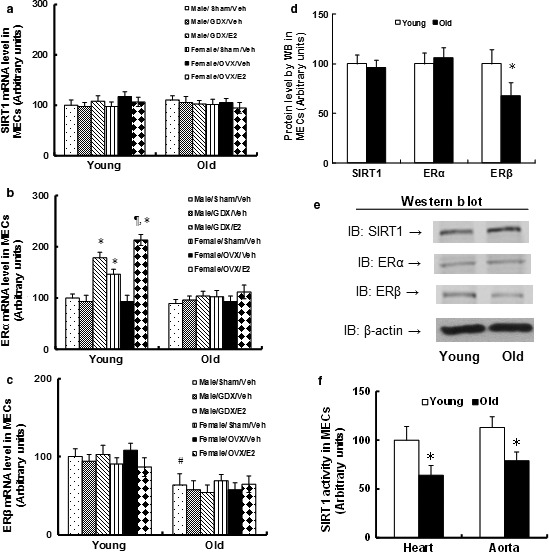
ERβ expression reduces in the endothelium from aging mice, while SIRT1 activity declines with no expression changes. The Young (6 months) and Old (30 months) mice received either sham, GDX (gonadectomy, for male), or OVX (ovariectomy, for female) surgery and were treated with either vehicle (Veh) or estradiol (E2) for 2 weeks. The mice were then sacrificed, and the MECs were isolated for analysis. (a–c) The MECs were isolated from treated mice using laser capture microdissection (LCM) techniques, and the mRNA was measured by qPCR. (a) SIRT1 mRNA level; (b) ERα mRNA level; (c) ERβ mRNA level. *n* = 5. *, *P *<* *0.05, vs. Male/Sham/Veh group; ¶, *P *<* *0.05, vs. Male/GDX/E2 group; #, *P *<* *0.05, vs. Male/Sham/Veh treatment in Young group. (d, e) The MECs were isolated for protein analysis by Western blotting. (d) The quantitative results for protein levels. (e) Representative Western blotting bands for (d), *n* = 5. *, *P *<* *0.05, vs. Young group. (f). The MECs were isolated from either the heart or aorta for measurement of SIRT1 activity. *n* = 5, *, *P *<* *0.05, vs. Young group. Results are expressed as mean ± SEM.

First, we measured the mRNA expression in mouse endothelial cells (MECs) that was isolated from thoracic aortas using laser capture microdissection (LCM) techniques. The results showed that the SIRT1 mRNA level did not change after either GDX/OVX or Veh/E2 treatment in both Young and Old mice (see Fig. 1a). The female mice had higher expressions of ERα than male mice, and the E2 treatment increased expression in Young mice (see Fig. 1b), while ERβ did not respond to the E2 treatment (see Fig. 1c). On the other hand, the basal expression of ERα did not change, while ERβ reduced significantly in Old mice compared to Young mice. This indicates that ERβ may play a dominant role in aging‐mediated vascular dysfunction, which is consistent with our recent finding showing that ERβ expression in the endothelium ameliorates ischemia/reperfusion‐mediated oxidative burst and vascular injury (Zhan *et al*., 2016). In order to investigate the role of ERβ in aging‐mediated vascular dysfunction, we chose male mice as the major animal model. If we had used female mice as the animal model, we would have had to do ovariectomy (OVX) to remove E2 because ERα is responsive to E2. The OVX would then be too artificial to the natural mice as OVX also removes other hormones, such as progesterone. We then measured protein levels in MECs isolated from the heart (See Fig. 1d,e). We found that the SIRT1 and ERα proteins did not change, while the ERβ reduced significantly in Old MECs. Finally, we measured the SIRT1 activity in MECs isolated from both the heart and aorta and found that the SIRT1 activity was decreased significantly in Old mice compared to Young mice (see Fig. 1f).

### SIRT1 induces ERβ expression through ‐254/‐235 PPRE site on the ERβ promoter

**Figure 2:**
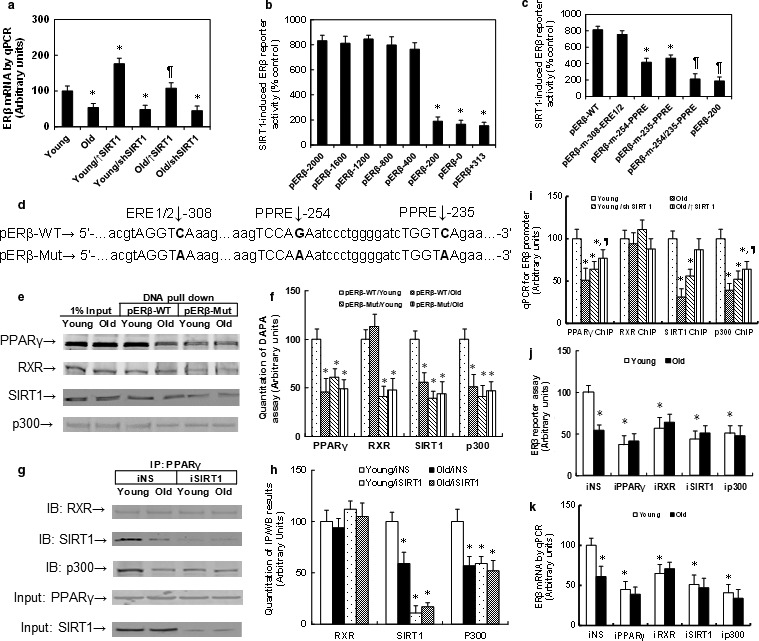
ERβ is regulated by SIRT1‐PPARγ/RXR‐p300 complexes through ‐254/‐235 PPRE site on the ERβ promoter. (a) The MECs were isolated from either Young or Old mice, and then treated by SIRT1 expression (↑SIRT1) or knockdown (shSIRT1) lentivirus, and the ERβ mRNA was measured by qPCR. *n* = 4, *, *P *<* *0.05, vs. Young group; ¶, *P *<* *0.05, vs. Old group. (b, c) The conditionally immortalized MECs were infected by either control or SIRT1 expression lentivirus, and the different ERβ deletion or mutant reporter plasmids were transfected. The SIRT1 expression‐induced (% control) luciferase activity was measured. (b) *n* = 4. *, *P *<* *0.05, vs. pERβ‐2000 group; (c) *n* = 4. *, *P *<* *0.05, vs. pERβ‐WT group; ¶, *P *<* *0.05, vs. pERβ‐m‐254‐PPRE group. (d) Schematic model for ERβ mouse promoter and mutation structure. (e) The biotin‐labeled oligonucleotides (fragment of ‐330~‐200 on ERβ promoter) were used for DNA pull‐down assay in the nuclear extracts from treated MECs, and the pull‐down proteins were blotted by PPARγ, RXR, SIRT1, and p300, respectively. (f) The quantitative results for e. *n* = 5. *, *P *<* *0.05, vs. pERβ‐WT/Young group. (g) The nuclear extracts from the above treated MECs were immunoprecipitated (IP) by PPARγ, then immunoblotted by RXR, SIRT1, p300, and PPARγ respectively, and 5% of input was blotted for PPARγ and SIRT1. (h) The quantitative results for g. *n* = 5. *, *P *<* *0.05, vs. Young/iNS group. (i) ChIP analysis on ERβ promoter using antibodies of PPARγ, RXR, SIRT1, and p300 respectively. *n* = 5. *, *P *<* *0.05, vs. Young group; ¶, *P *<* *0.05, vs. Old group. (j) The conditionally immortalized MECs were transfected by siRNA for NS (nonsense) control, PPARγ, RXR, SIRT1, and p300, together with the ERβ reporter plasmid, and the luciferase activity was measured. *n* = 5. *, *P *<* *0.05, vs. Young group. (k) The cells were treated by siRNA, and the mRNA level was measured. *n* = 5. *, *P *<* *0.05, vs. Young group. Results are expressed as mean ± SEM.

The MECs from Young and Old mice were isolated and treated using either SIRT1 expression (↑SIRT1) or knockdown (shSIRT1) lentivirus. As shown in Fig. 2a, ERβ expression decreased in Old MECs compared to Young MECs, and SIRT1 expression increased, while SIRT1 knockdown decreased ERβ expression. We investigated the molecular mechanism for SIRT1‐induced ERβ expression in endothelial cells. A series of progressive 5′‐promoter deletion constructs was generated according to the Ensembl ID: ENSMUST00000101291, and those constructs were transfected into immortalized MECs for the reporter activity assay. We found that the SIRT1‐induced ERβ transcriptional element is located in the range of −400 ~ −200 on the ERβ promoter (see Fig. 2b). The transcription factor database TESS revealed several potential binding motifs, including an ERE1/2 and a nontypical PPRE (PPAR response element) binding site. The single‐mutant reporter assay showed that the PPRE double mutants (pERβ‐m‐254/235‐PPRE) completely abolished the SIRT1 effect (see Fig. 2c,d), indicating that the ‐254/235‐PPRE element in the range of −400 ~ −200 is required for SIRT1‐induced ERβ activation. The DAPA assay was used to measure the binding abilities of those transcription factors to the ERβ promoter, as shown in Fig. 2e,f. The results showed that the PPARγ, SIRT1, and p300 had decreased binding, and RXR had no effect on ERβ wild‐type (WT) promoter (pERβ‐WT) in Old MECs compared to Young MECs. All the above transcription factors showed decreased binding to the PPRE mutation promoter (pERβ‐Mut, see Fig. 2d). This indicates that the PPRE element is responsible for SIRT1‐mediated ERβ expression. We then used an immunoprecipitation assay to measure the interaction of those transcription factors. In Fig. 2g,h, the PPARγ had decreased interaction with SIRT1 and p300 in Old MECs compared to Young MECs, while in SIRT1 knockdown MECs, PPARγ had decreased interaction with p300 in Young MECs, mimicking the effect of Old MECs. This indicates that SIRT1 plays an important role in the interaction of PPARγ and p300 (see Fig. 2g,h). In Fig. 2i, the ERβ promoter was precipitated with ChIP (chromatin immunoprecipitation) techniques using antibodies for PPARγ, RXR, SIRT1, and p300, respectively. Results showed that the Old MECs have decreased association with PPARγ, SIRT1, and p300 on the ERβ promoter compared to Young MECs, while the SIRT1 overexpression (↑SIRT1) increased, and the SIRT1 knockdown (shSIRT1) decreased association of those factors to the ERβ promoter. On the other hand, the RXR did not change. Finally, the siRNA technique was used to knockdown those transcription factors to investigate the potential contribution to ERβ expression. From both the ERβ reporter assay (see Fig. 2j) and mRNA expression assay (see Fig. 2k), we found that knockdown of any one of those factors significantly decreased ERβ activation, mimicking the effect of Old MECS with decreased ERβ expression.

### Reduced ERβ expression is due to compromised phosphorylation of amino acid S154 in SIRT1, and single‐mutant SIRT1‐C152(D) restores this effect in aging mice

**Figure 3:**
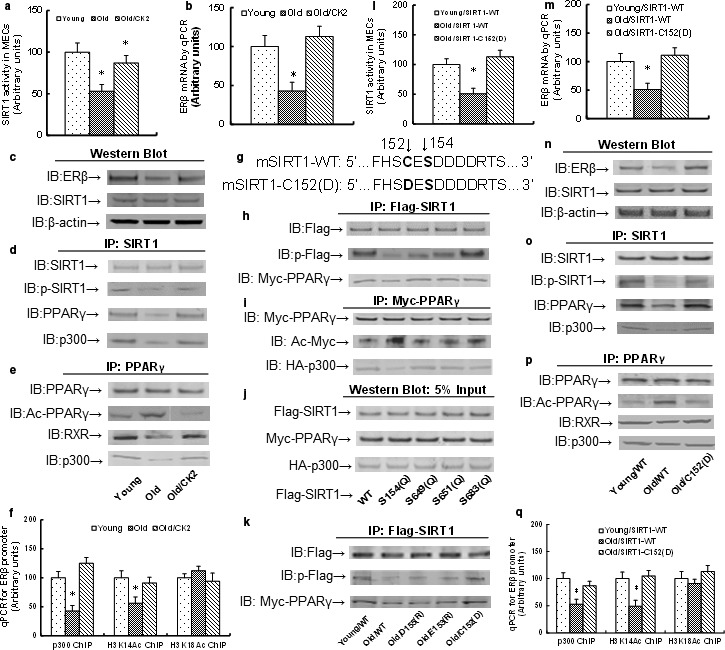
Reduced ERβ expression in the endothelium from aging mice is due to compromised phosphorylation of amino acid S154 in SIRT1, and the mutant SIRT1‐C152(D) restores this effect. (a–f) The MECs from either Young or Old mice were infected by either control or CK2 lentivirus, and the cells were harvested for further analysis. (a) SIRT1 activity. (b) ERβ mRNA. (c) Protein analysis by Western blotting. (d) IP/WB analysis for SIRT1. (e) IP/WB analysis for PPARγ. (f) ChIP analysis on ERβ promoter. *n* = 4. *, *P *<* *0.05, vs. Young group. (g) Schematic model for mouse single‐mutant mSIRT1‐C152(D). (h–j) The conditionally immortalized Young MECs were transfected by either Flag‐SIRT1 WT (wild‐type) or single mutants for further analysis. (h) IP/WB analysis of Flag‐SIRT1. (i) IP/WB analysis of Myc‐PPARγ. (j) Western blots analysis of 5% input. (k) The conditionally immortalized MECs from Young or Old mice were transfected by either Flag‐SIRT1 WT (wild‐type) or single mutants for IP/WB analysis. (l–q) The MECs from either Young or Old mice were infected by either SIRT1‐WT or single‐mutant C152(D) lentivirus, and the cells were harvested for further analysis. (l) SIRT1 activity. (m) ERβ mRNA. (n) Protein analysis by Western blotting. (o) IP/WB analysis for SIRT1. (p) IP/WB analysis for PPARγ. (q) ChIP analysis on ERβ promoter. *n* = 4. *, *P *<* *0.05, vs. Young group. Results are expressed as mean ± SEM.

The isolated MECs were infected by either an empty or CK2 (casein kinase 2) lentivirus for further analysis. In Fig. S1a, CK2 activity was decreased in Old MECs compared to Young MECs, while the activity increased more than fourfold in Old MECs after CK2 lentivirus infection. We then measured the molecular consequences of CK2 expression and found that the declined SIRT1 activity (see Fig. 3a), decreased ERβ mRNA (see Fig. 3b), and decreased protein (see Fig. 3c) were completely restored by CK2 expression in Old MECs compared to Young MECs, while the SIRT1 protein did not change (see Fig. 3c and Fig. S1b). This indicates that CK2‐mediated phosphorylation may be involved in declined SIRT1 activity in Old MECs. In Fig. 3d and Fig. S1c (Supporting information), the SIRT1 had compromised phosphorylation and decreased association with PPARγ and p300 in Old MECs, and this effect was completely restored by CK2 expression. In Fig. 3e and Fig. S1d, the PPARγ had increased acetylation and decreased association with p300 in Old MECs, which was restored by CK2 expression. In Fig. 3f, the p300 had decreased binding to the ERβ promoter, and the epigenetic changes on the ERβ promoter showed decreased acetylation on H3K14 instead of H3K18 in Old MECs. This effect was again restored by CK2. These results indicate that the compromised SIRT1 phosphorylation in Old MECs results in decreased association of SIRT1 with PPARγ. This leads to increased PPARγ acetylation and decreased association with p300, which then subsequently leads to decreased H3K14 acetylation on the ERβ promoter with decreased ERβ expression. In order to map the ERβ‐responsive SIRT1 phosphorylation site, the SIRT1 wild‐type (WT) or single mutants as indicated (see Fig. 3g) were transfected into immortalized MECs for IP/WB analysis. In Fig. 3h,j and Fig. S1e,g, the mutants of S154(Q), S649(Q), and S651(Q) significantly decreased the total phosphorylation. The S683(Q) mutant had no effect, while only the mutant S154(Q) significantly decreased the association of SIRT1 with PPARγ. In Fig. 3i,j and Fig. S1f,g, the single‐mutant S154(Q) significantly increased PPARγ acetylation and decreased association of PPARγ with p300. This indicates that S154 phosphorylation may play a dominant role in ERβ regulation. We then generated several single mutants that were close to the major phosphorylation site S154 for IP/WB analysis. In Fig. 3k and Fig. S1h, the single‐mutant C152(D) significantly restored the SIRT1 phosphorylation in Old MECs, and it also restored the association of SIRT1 with PPARγ. Furthermore, we made 11 SIRT1 single mutants for IP/WB analysis of SIRT1 phosphorylation and ERβ mRNA expression as shown in Table S3 (Supporting information). We found that both the phospho‐preventive alanine mutation (S154A) and the phosphomimetic mutation (S154D or S154E) reduced the SIRT1 phosphorylation and ERβ mRNA expression. We also made a polar (hydrophilic) mutation to S154Q, and it could not restore aging‐mediated reduced SIRT1 phosphorylation and ERβ expression as well. Finally, we made many mutations from proxy amino acids, including D155R, E153R, D156R, and C152R, and none of them worked, but rather made it worse. From these experiments, we found that the proxy negatively charged amino acid D or E seems to favor the phosphorylation of amino acid S. If we made a proxy mutation from negatively charged D or E to positively charged R, the S154 phosphorylation would be diminished. We then made proxy amino acid C152 mutation to negatively charged C152D or C152E, and the results showed that both of them significantly increased the SIRT1 phosphorylation and ERβ expression, and the mutant C152D showed better effects than C152E. That was why we chose C152D for further experiments to restore aging‐mediated reduced SIRT1 phosphorylation and ERβ expression. As the crystallization structure of SIRT1 is still unavailable, we suppose that the single‐mutant SIRT1‐C152(D), from polar amino acid cysteine(C) to strongly acidic amino acid aspartic acid(D), changes the structure of proxy target S154, so S154 can be more easily phosphorylated, while it is also possible that the SIRT1‐C152D mutant is simply more biochemically active. This is the first time that we have developed a novel method to restore the target amino acid function through a proxy single mutant. We found that SIRT1 single‐mutant C152D significantly restored the SIRT1 phosphorylation and ERβ expression in Old MECs. This indicates that the single‐mutant SIRT1‐C152(D) may prevent Old MECs‐mediated compromised S154 phosphorylation, reduced ERβ expression, and the subsequent endothelial dysfunction. Finally, the SIRT1 lentivirus for wild‐type (WT) or single‐mutant C152(D) was generated and the MECs were infected to evaluate their subsequent cellular function. In Fig. 3l, expression of SIRT1 wild‐type (SIRT1‐WT) had decreased SIRT1 activity in Old MECs compared to Young MECs, while the single‐mutant SIRT1‐C152(D) expression in Old MECs restored this effect, and also restored the ERβ mRNA (see Fig. 3m) and protein expression (see Fig. 3n and Fig. S1i). Furthermore, SIRT1‐C152(D) expression partly restored the compromised SIRT1 phosphorylation and decreased association of SIRT1 with PPARγ and p300 (see Fig. 3o and Fig. S1j) in Old MECs. It completely restored Old MECs‐mediated increased PPARγ acetylation and decreased association of PPARγ with p300 (see Fig. 3p and Fig. S1k), and also restored Old MECs‐mediated decreased binding of p300 to the ERβ promoter and decreased H3K14 acetylation on the ERβ promoter (see Fig. 3q). These results further prove that the single‐mutant SIRT1‐C152(D) restores Old MECs‐mediated ERβ suppression.

### Expression of ERβ and single‐mutant SIRT1‐C152(D) restores the expression of ERβ target genes in the endothelium from aging mice

**Figure 4:**
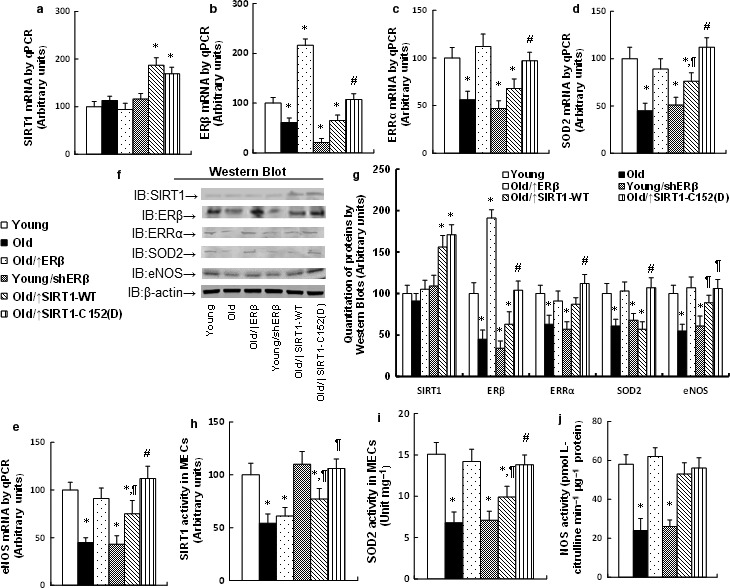
Expression of ERβ and the mutant SIRT1‐C152(D) restores the expression of ERβ target genes in the endothelium from aging mice. (a–e) The MECs were isolated from the heart using laser capture microdissection techniques to measure mRNA level by qPCR for SIRT1 (a), ERβ (b), ERRα (c), SOD2 (d), and eNOS (e), *n* = 4. (f–g) The isolated MECs from the heart were used for protein analysis by Western blotting. (f) Representative bands by Western blots. (g) Protein quantitative results for (f), *n* = 4. (h–j) The isolated MECs were used for the enzyme activity assay of SIRT1 (h), SOD2 (i), and NOS (j). *n* = 5. *, *P *<* *0.05, vs. Young group; ¶, *P *<* *0.05, vs. Old group; #, *P *<* *0.05, vs. Old/↑SIRT1‐WT group. Results are expressed as mean ± SEM.

The MECs were isolated from the heart in treated mice using laser capture microdissection techniques for mRNA analysis. In Fig. 4a, the SIRT1 mRNA did not change in either Young or Old MECs, while the SIRT1 lentivirus infection significantly increased the expression. In Fig. 4b, the ERβ lentivirus infection significantly increased ERβ expression in Old MECs, and the shERβ lentivirus decreased ERβ expression in Young MECs. The SIRT1‐WT expression did not change, while the single‐mutant SIRT1‐C152(D) expression restored the ERβ expression in Old MECs. We then measured the ERβ target genes, including ERRα (see Fig. 4c), SOD2 (see Fig. 4d), and eNOS (see Fig. 4e). We found that those genes were decreased in Old MECs, and the expression of ERβ restored this effect, while ERβ knockdown by shERβ in Young MECs decreased their expression. On the other hand, the SIRT1‐WT expression showed little effect, while the single‐mutant SIRT1‐C152(D) expression completely restored the gene expression. We further confirmed the effect for those gene expression changes through Western blotting using *in vitro* cultured MECs (see Fig. 3f,g). We also measured the enzyme activities for those genes. In Fig. 4h, SIRT1 activity declined in Old MECs and was not affected by ERβ expression. Expression of SIRT1‐WT in Old MECs significantly increased its activity, but it was still lower than the activity in Young MECs. On the other hand, the single‐mutant SIRT1‐C152(D) expression completely restored the SIRT1 activity in Old MECs. We also measured the enzyme activities for SOD2 (see Fig. 4i) and NOS (see Fig. 4j). The activities were similar with their expression levels and were regulated by ERβ expression. SIRT1‐WT expression showed little effect, while SIRT1‐C152(D) expression significantly restored their activities. We then measured the gene expression of ERβ (see Fig. S2a) and SIRT1 (see Fig. S2b) in isolated mouse endothelial cells (MECs) and mouse cardiomyocytes (MCMs) from the hearts using laser capture microdissection techniques. The results showed that Tie2‐driven lentivirus infection was only expressed in MECs, but not in MCMs. We also measured the gene expression of lentivirus‐carrying SIRT1 wild‐type SIRT1‐WT and single‐mutant SIRT1‐C152(D) (see Fig. S3). Two pairs of specific primers were designed to distinguish the one amino acid difference from 152C (coded by TGT) to 152D (coded by GAT). In Fig. S3a, the mRNA expression of SIRT1‐WT increased around 1.9‐fold in the Old/↑SIRT1‐WT group compared to other groups. In Fig. S3b, the mRNA of SIRT1‐C152(D) was detected only in the Old/↑SIRT1‐C152(D) group, and they were not detectable from other groups. This indicates that the lentivirus‐carrying expression for SIRT1 wild‐type or single‐mutant SIRT1‐C152(D) was efficient and specific. We then measured the gene expression of ERβ (see Fig. S4a) and SIRT1 (see Fig. S4b) from other whole tissues, including the liver, kidney, and hypothalamus. The whole tissues had no expression difference, and interestingly, the ERβ expression was reduced in the liver and kidney from old mice. We also investigated the effects of Tie2‐driven lentivirus on the myeloid‐derived endothelial progenitor cells (EPCs), as shown in Fig. S5. The EPCs and the related control (CTL) cells were isolated for mRNA measurement. We found that the mRNA expression of ERβ (see Fig. S5a) and SIRT1 (see Fig. S5b) in EPCs did not change in Young and Old mice, and interestingly, the lentivirus infection did not affect the gene expression at all. This can be explained with how the Tie2‐driven lentivirus infection through tail vein injection mostly targets the endothelium on the vascular wall, and it has too few viruses that could reach the myeloid to reflect significant expression changes in the myeloid. In general, we showed that Tie2‐driven lentivirus infection in the endothelium did not directly affect the gene expression in those whole tissues, indicating that our lentivirus infection was specific, with no obvious leaking effects.

### Expression of ERβ and the mutant SIRT1‐C152(D) minimizes ROS generation and DNA damage in the endothelium from aging mice

**Figure 5:**
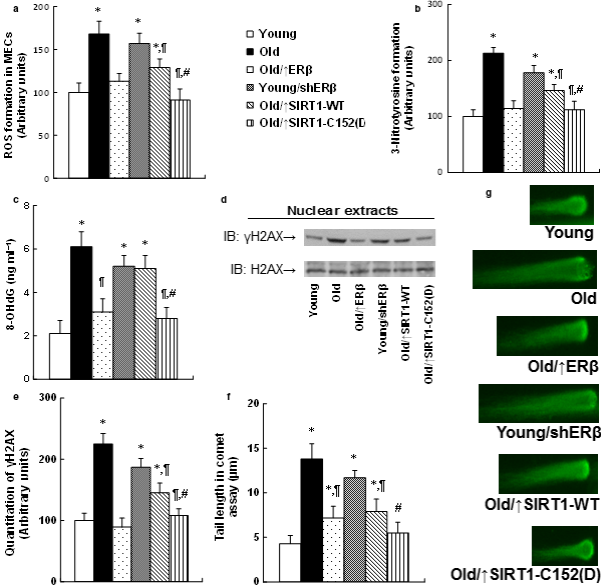
Expression of ERβ and the mutant SIRT1‐C152(D) minimizes ROS generation and DNA damage in the endothelium from aging mice. The MECs were isolated from the hearts with indicated treatments for further analysis. (a) ROS formation. (b) Quantitation of 3‐nitrotyrosine (3‐NT) formation. (c) 8‐OHdG formation. (d) Representative γH2AX Western blotting band. (e) Quantitation of γH2AX formation for (d). (f) Quantitation of Tail length in comet assay. (g) Representative picture for (f). *n* = 5. *, *P *<* *0.05, vs. Young group; ¶, *P *<* *0.05, vs. Old group; #, *P *<* *0.05, vs. Old/↑SIRT1‐WT group. Results are expressed as mean ± SEM.

We measured the ROS generation and some marks for DNA damage. The ROS generation (see Fig. 5a) and the 3‐nitrotyrosine (3‐NT) formation (see Fig. 5b) were increased in Old MECs, and they were minimized by ERβ expression, but increased by shERβ. Expression of SIRT1‐WT partly restored this effect, while SIRT1‐C152(D) expression completely restored it. We also measured the 8‐OHdG generation (see Fig. 5c), γH2AX formation (see Fig. 5d,e), and tail length in comet assay (see Fig. 5f,g). Similarly, the Old MECs‐mediated DNA damage was minimized by ERβ expression, worsened by shERβ, partly restored by SIRT1‐WT expression, but completely restored by single‐mutant SIRT1‐C152(D) expression.

### Expression of ERβ and the mutant SIRT1‐C152(D) restores mitochondrial dysfunction in the endothelium from aging mice

We measured the effects of the mitochondrial function, including mitochondrial DNA copies (see Fig. S6a), caspase‐3 activity (see Fig. S6b), intracellular ATP level (see Fig. S6c), mitochondrial mass (see Fig. S6d), mitochondrial membrane potential (Δψm, see Fig. S6e), and mitochondrial OXPHOS proteins (see Fig. S6f,g). Our results showed that ERβ expression increased, while shERβ decreased mitochondrial function. The SIRT1‐WT expression had little effect on the mitochondrial function, and the SIRT1‐C152(D) expression completely restored the Old MECs‐mediated mitochondrial dysfunction.

### Expression of ERβ and the mutant SIRT1‐C152(D) restores dysfunction of fatty acid metabolisms in the endothelium from aging mice

We measured the *in vitro* fatty acid uptake (see Fig. S7a) and oxidation (see Fig. S7b). The results showed that the expression of ERβ and SIRT1‐C152(D) completely restored Old MECs‐mediated decreased fatty acid metabolism, instead of SIRT1‐WT expression, and the ERβ knockdown (shERβ) worsened the problem. Finally, we measured the *in vivo* fatty acid metabolism using mice infected by the indicated lentivirus through tail vein injection. Our results showed that the SIRT1‐WT expression had little effect, while the expression of ERβ and SIRT1‐C152(D) significantly increased fatty acid uptake in both the heart and aorta (see Fig. S7c) with decreased plasma fatty acid level (see Fig. S7d), while there was no effect on the liver (see Fig. S7e). This indicates that SIRT1‐C152(D) expression in the endothelium from aging mice can increase the *in vivo* fatty acid metabolism and reduce the circulating lipids.

### Expression of ERβ and single‐mutant SIRT1‐C152(D) reduces circulating lipids and ameliorates vascular damage in the endothelium from aging mice

**Figure 6:**
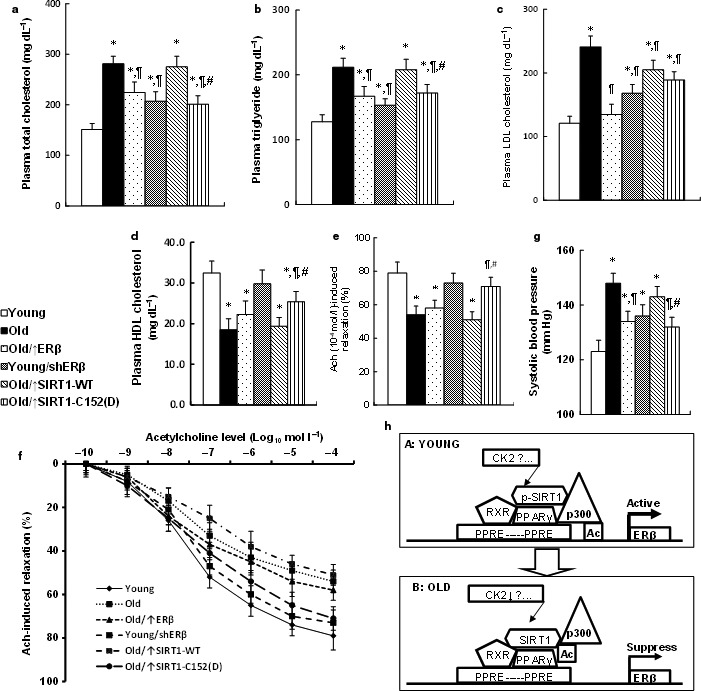
Expression of ERβ and the mutant SIRT1‐C152(D) on the vascular wall reduces circulating lipids and ameliorates vascular damage in aging mice. (a–d) The plasma was collected from treated mice for analysis of total cholesterol, *n* = 11 (a); triglyceride, *n* = 9 (b); LDL cholesterol, *n* = 11 (c); and HDL cholesterol, *n* = 9 (d). (e, f) The aortas were dissected from treated mice for vessel tension analysis. The rings were preconstricted with phenylephrine, and the acetylcholine (Ach, 10^−10^–10^−4^ mol/L) was injected at the plateau of the phenylephrine‐induced contraction. (e) The 10^−4^ mol/L Ach‐induced aorta ring relaxation, *n* = 11; (f) The Ach‐induced aorta ring relaxation curves, *n* = 12. (g) The treated mice were used to measure the mean of systolic blood pressure, *n* = 10. (h) Schematic model for SIRT1‐mediated ERβ suppression in vascular aging. *, *P *<* *0.05, vs. Young group; ¶, *P *<* *0.05, vs. Old group; #, *P *<* *0.05, vs. Old/↑SIRT1‐WT group. Results are expressed as mean ± SEM.

In order to evaluate the effects of SIRT1‐C152(D) expression in the endothelium on the vascular function, we measured the plasma lipid levels (see Fig. 6a–d). Old mice had significantly increased plasma lipid levels, including total cholesterol (see Fig. 6a), triglyceride (see Fig. 6b), and LDL cholesterol (see Fig. 6c), but decreased HDL cholesterol level (see Fig. 6d). ERβ expression in Old mice partly restored this effect, while shERβ expression worsened the problem. SIRT1‐WT expression showed little effect, while SIRT1‐C152(D) expression significantly restored the plasma lipids. We then measured the effect of SIRT1‐C152(D) expression on the changes of vessel tension. In Fig. 6e,f, the acetylcholine‐induced relaxation was significantly decreased in Old mice compared to Young mice, and this effect was partly restored by expression of ERβ and SIRT1‐C152(D) in Old mice, worsened by shERβ expression in Young mice, while showing no effect by expression of SIRT1‐WT. We also measured the effect of SIRT1‐C152(D) expression on the changes of blood pressure. As shown in Fig. 6g, the blood pressure was increased and was significantly restored by ERβ or SIRT1‐C152(D) expression in Old mice, but worsened by shERβ expression in Young mice, while there was little effect from SIRT1‐WT expression. Finally, we have also briefly repeated experiments in female mice similar to the ones we did in male mice (see Fig. S8). We found that the Tie2‐driven lentivirus infection through tail vein injection is efficient for manipulating the ERβ expression on the vascular wall (see Fig. S8a). Also, the expression of either ERβ or SIRT1‐S152(D) significantly ameliorated aging‐mediated hypertension in Old mice (see Fig. S8b) and shows a similar effect in male mice. This suggests that ERβ may play a similar role in vascular aging for both male and female mice. In addition, our results showed that the expression of ERβ or single‐mutant SIRT1‐C152(D) in the endothelium could only partly restore aging‐mediated vascular dysfunction. This suggests that the SIRT1 may function through another pathway in addition to the ERβ pathway, or that another totally different factor is involved in this process for vascular aging.

We have shown a schematic model to explain the mechanism for SIRT1‐mediated ERβ suppression in vascular aging. In Young mice, the SIRT1 is activated by some kinase (e.g. CK2) through phosphorylation, especially at the site of S154, and then the activated SIRT1 binds with and deacetylates PPARγ. The acetylated PPARγ increases the association with p300, and the complexes of SIRT1‐PPARγ/RXR‐p300 show increased binding ability to the ERβ promoter through the PPRE site. The recruitment of p300 results in increased acetylation of H3K14 on the ERβ promoter and subsequently activates the ERβ expression (see Fig. 6h, panel A). On the other hand, in Old mice (see Fig. 6h, panel B), the SIRT1 is inactivated by declined phosphorylation at the S154 site, which results in decreased PPARγ binding and increased PPARγ acetylation, so that the complexes of SIRT1‐PPARγ/RXR‐p300 have decreased binding to the ERβ promoter. Therefore, the decreased p300 recruitment cannot acetylate the H3K14 on the ERβ promoter, which eventually leads to ERβ suppression.

## Discussion

### Declined SIRT1 activity in vascular aging

Phosphorylation has been reported to be the major post‐translational modification (Bai *et al*., 2012; Flick & Luscher, 2012), and the protein kinase CK2 phosphorylation sites have been identified in murine SIRT1, including S154, S649, S651, and S683 (Kang *et al*., 2009). Given the fact that the CK2 activity was decreased in aging mice, we then hypothesize that decreased CK2 activity in aging mice may contribute to the declined SIRT1 phosphorylation and subsequent declined SIRT1 activity and reduced ERβ expression, even though we cannot rule out other kinds of kinase or factors, which may contribute to the SIRT1 phosphorylation as well. In our study, the major reason why we used CK2 overexpression was to restore aging‐induced decreased SIRT1 phosphorylation (Kang *et al*., 2009). The two sites of S649/S651 in murine SIRT1 have been identified in humans (Zschoernig & Mahlknecht, 2009) and found to be the ESA (essential for SIRT1 activity) motif (Kang *et al*., 2011). We did further investigation on those sites, and the mutants of S649/651(Q) did not show significant functional differences. On the other hand, the mutant S154(Q) in SIRT1 significantly decreased the association of SIRT1 with PPARγ. It also increased PPARγ acetylation and decreased association of PPARγ with p300. This indicates that the phosphorylation of S154 in SIRT1 plays an essential role in ERβ regulation.

### SIRT1‐mediated ERβ expression

Our preliminary experiments showed that ERβ mRNA expression was significantly reduced in Old mice compared to Young mice, while ERα expression did not change. We further measured the protein expression from *in vitro* cultured MECs to confirm the changes, and it showed that ERβ expression was reduced, while ERα had no changes. These data indicate that the expression of ERβ is regulated by aging‐mediated factors, while ERα is not. Our further experiments showed that the ERβ was regulated by SIRT1, while the ERα was not responsive to the SIRT1 expression. We then tried to map the aging‐responsive element in the ERβ promoter using the luciferase reporter assay. Our results showed that the PPRE element is responsible for the aging‐mediated reduced ERβ expression. Further investigation using DAPA assay (DNA pull‐down assay) showed that the PPARγ, RXR, p300, and SIRT1 are binding to the PPRE element (and/or proxy sequence) on the ERβ promoter, and the ChIP assay also showed that SIRT1 is binding to the PPRE element as well. These results indicate that SIRT1 regulates the ERβ expression. We then measured SIRT1 expression and activity and found that the SIRT1 expression was not changed, but the activity was declined. Given the fact that many literatures report that the SIRT1 is involved in the aging process, we then focused on the sirtuin pathway as a mechanism for aging‐mediated ERβ regulation.

We have identified a novel mechanism for SIRT1‐mediated ERβ regulation through the complexes SIRT1‐PPARγ/RXR‐p300 that bind to the PPRE site on the ERβ promoter. Our results showed that the identified PPRE site is slightly different from the typical PPRE sequence (Tugwood *et al*., 1992), and our preliminary data from the DAPA assay and ChIP assay showed that the SIRT1‐related transcription factors, including AMPK (Price *et al*., 2012), PGC1α, and PPARα (Oka *et al*., 2011) did not bind to our identified PPRE site on the ERβ promoter. This indicates that they are not directly involved in SIRT1‐mediated ERβ expression. We have shown that the phosphorylated SIRT1 binds with and deacetylates PPARγ (Qiang *et al*., 2012), and the deacetylated PPARγ then interacts with RXR as a heterodimer, binds to the PPRE site (Schulman *et al*., 1998), and recruits p300 (Gelman *et al*., 1999). Subsequently, p300 binds to the promoter and acetylates the H3K14 (Schiltz *et al*., 1999), and eventually activates the ERβ expression. In this study, the PPARγ deacetylation by SIRT1 seems to be activating instead of suppressing PPARγ in the endothelium (Picard *et al*., 2004). In order to investigate p300‐mediated epigenetic histone acetylation on the ERβ promoter, we have tried sites for H3K14, H3K18, H4K5, and H4K8, but only H3K14 was positive, indicating that p300 regulates the ERβ expression by modification of H3K14 acetylation in the endothelium from aging mice.

### SIRT1/ERβ‐mediated vasculoprotection in the endothelium from aging mice

In order to evaluate the vasculoprotective effect of SIRT1/ERβ expression in the endothelium, the Tie2‐driven lentivirus was used to deliver the SIRT1/ERβ on the vascular wall. Given the fact that the Tie2‐driven expression has a potential leaking effect, especially on hematopoietic lineages (Favre *et al*., 2010), we measured the gene expression from other tissues. The results showed that the ERβ/SIRT1 expression had no changes on the liver, kidney, hypothalamus (see Fig. S4), and myeloid‐derived EPCs (see Fig. S5), indicating that there is no obvious leaking effect. Also, the specificity of the lentivirus infection has been discussed in detail previously (see Figs S2 and S3), and this approach meets our experimental requirements (Li *et al*., 2015). We have previously reported that ERβ is responsible for the basal expression of SOD2 and ERRα in the endothelium in the absence of estrogen (Liu *et al*., 2014; Li *et al*., 2015). In this study, the Old MECs with the expression of ERβ or SIRT1‐C152(D) showed minimized ROS generation and DNA damage. This can be explained by SIRT1‐C152(D)/ERβ‐mediated SOD2 upregulation (Liu *et al*., 2014). On the other hand, the Old MECs with the expression of ERβ or SIRT1‐C152(D) showed increased mitochondrial function and fatty acid metabolism, and this can be explained by SIRT1‐C152(D)/ERβ‐mediated ERRα upregulation (Li *et al*., 2015). Furthermore, the *in vivo* experiments with lentivirus‐carrying ERβ or SIRT1‐C152(D) expression on the vascular wall showed reduced circulating lipids with ameliorated vascular damage. This proves that ERβ and SIRT1 in the endothelium play an essential role in vascular aging.

Our results showed that the effect of SIRT1 on vascular function can be regulated through modulation of ERβ expression, and ERβ will then modulate the vascular function through its target genes, and eventually contributes to vascular aging. On the other hand, the SIRT1 can also act through a mechanism different from ERβ. For instance, SIRT1 activity can be regulated by Ca2^+^/calmodulin‐dependent protein kinase kinase (CaMKK)β (Wen *et al*., 2013), and SIRT1 can function through FoxO, PPARγ, PGC1α, AMPK, and NF‐κB (Potente & Dimmeler, 2008). Also, SIRT1 can deacetylate eNOS, stimulate the activity, and increase NO production (Mattagajasingh *et al*., 2007). This is consistent with our observation that the expression of ERβ or SIRT1 single‐mutant C152(D) can only partly restore aging‐mediated vascular dysfunction, indicating that some other factors may also be involved in this process.

## Experimental procedures

An expanded Experimental Procedures section is available in Data S1 (Supporting information).

### *In vivo* mouse experiments

To investigate the effect of lentivirus‐carrying gene expression on vascular aging, the male mice (C57BL/6J) fed with high‐fat diet (HFD, 60% calories from fat, Research Diets, #D12492) were used in this study throughout the experiments. The Young (4 months) and Old (28 months) mice received tail vein injections of 150 μL of lentivirus (2 × 10^8^ MOI) for the Tie2‐Empty (CTL), Tie2‐↑ERβ, Tie2‐↑SIRT1, or Tie2‐shERβ twice within a two‐day interval. This injection procedure was administered again after 1 month. After 2 months, the Young (6 ms) and Old (30 ms) mice were overnight‐fasted, euthanized by 100 mg kg^−1^ pentobarbital, and the blood was collected for measuring plasma ^14^C‐OA, estrogen (E2), and lipids, including total cholesterol, triglyceride, LDL, and HDL cholesterol. The MECs from the heart were isolated for *in vitro* cell culture analysis, and the MECs from the thoracic aortas were picked up by laser capture microdissection (LCM) for mRNA analysis. The carotid arteries were isolated to measure vessel tension. In some treatments, the hearts were dissected and snap‐frozen in the OCT compound. The 10‐μm sections were cut using a clean microtome and mounted on PEN‐membrane slides (2.0 μm, Leica, Wetzlar, Germany) for isolation of mouse endothelial cells (MECs) and mouse cardiomyocytes (MCMs) using laser capture microdissection (LCM) for mRNA analysis. Also, in some experiments, the female OVX mice were used to briefly repeat the same experiments as the ones done on male mice.

## Author contributions

P.Y., Y.D., and ZX.L. designed, interpreted the experiments, and wrote the paper. D.K. H.Y., H.L., and Y.D. performed the mapping experiments. ZY.L.,T.D., A.L., S.W., and A.L. performed the estrogen treatment and LCM techniques. Y.Z., M.L., D.Z., Y.W., H.Z., Z.Z., X.H., P.Y., and ZX.L. performed the *in vivo* mice experiments. D.K., Y.Z., and ZY.L. performed the remaining experiments.

## Funding

This study was financially supported by The National Natural Science Foundation of China, Project #81273166; The International Technology Cooperation Projects of Dongguan (#:20135081520017); The Features Innovative Projects of Key Platform and Major Scientific Research Project of Universities in Guangdong Province (#:2015KTSCX048); Hubei Science & Technology Development Project # 2014CFB437; and The 2013 International Science & Technology Cooperation Project of Ministry of Education (China) #: 2013DFA31400.

## Conflict of interest

None.

## Supporting information


**Data S1** Experimental procedures.
**Table S1** Sequences of primers for the real time quantitative PCR (qPCR)
**Table S2** Details and conditions for the mice treatment
**Table S3** Effects of C152, E153, S154, D155 and D156 mutations on the SIRT1 phosphorylation and ERβ expression in MECs cells
**Fig. S1** Reduced ERβ expression is due to compromised phosphorylation at aa S154 in SIRT1, the mutant SIRT1‐C152(D) restores this effect in the endothelium from aging mice.
**Fig. S2** Tie2‐driven lentivirus expression is specific in the endothelium, instead of other cells.
**Fig. S3** Tie2‐driven lentivirus expression of SIRT1‐WT and SIRT1‐C152(D) is specific in the endothelium.
**Fig. S4** Tie2‐driven lentivirus expression is specific in the endothelium, instead of other tissues.
**Fig. S5** Tie2‐driven lentivirus infection through tail vein injection has no significant effect on myeloid EPCs.
**Fig. S6** Expression of ERβ and the mutant SIRT1‐C152(D) restores mitochondrial dysfunction in the endothelium from aging mice.
**Fig. S7** Expression of ERβ and the mutant SIRT1‐C152(D) restores dysfunction of fatty acid metabolisms in the endothelium from aging mice.
**Fig. S8** Expression of ERβ and the mutant SIRT1‐C152(D) on the vascular wall ameliorates vascular damage in aging female mice.
