# Supplementary material for: SIRT1‐mediated ERβ suppression in the endothelium contributes to vascular aging
Source: Aging Cell. 2016 Jul 29;15(6):1092–102. doi: 10.1111/acel.12515 (PMC6398526; doi:10.1111/acel.12515)
Supplement: Supplementary file 1 — Data S1 Experimental procedures. Table S1 Sequences of primers for the real time quantitative PCR (qPCR) Table S2 Details and conditions for the mice treatment Table S3 Effects of C152, E153, S154, D155 and D156 mutations on the SIRT1 phosphorylation and ERβ expression in MECs cells Fig. S1 Reduced ERβ expression is due to compromised phosphorylation at aa S154 in SIRT1, the mutant SIRT1‐C152(D) restores this effect in the endothelium from aging mice. Fig. S2 Tie2‐driven lentivirus expression is specific in the endothelium, instead of other cells. Fig. S3 Tie2‐driven lentivirus expression of SIRT1‐WT and SIRT1‐C152(D) is specific in the endothelium. Fig. S4 Tie2‐driven lentivirus expression is specific in the endothelium, instead of other tissues. Fig. S5 Tie2‐driven lentivirus infection through tail vein injection has no significant effect on myeloid EPCs. Fig. S6 Expression of ERβ and the mutant SIRT1‐C152(D) restores mitochondrial dysfunction in the endothelium from aging mice. Fig. S7 Expression of ERβ and the mutant SIRT1‐C152(D) restores dysfunction of fatty acid metabolisms in the endothelium from aging mice. Fig. S8 Expression of ERβ and the mutant SIRT1‐C152(D) on the vascular wall ameliorates vascular damage in aging female mice. [file ACEL-15-1092-s001.doc]

**SIRT1-mediated ERβ suppression in the endothelium contributes to vascular aging**

SUPPORTING INFORMATION

**Data S1: Experimental Procedures**

**Materials.** The antibodies for ERα (ab37438), ERRα (ab37438), H2AX (ab20669) and γH2AX (ab2893) were obtained from Abcam, antibodies for Acetyl-Histone H3 Lys9 (#9649), Lys14 (#7627) and Lys18 (#13998) were obtained from Cell Signaling, Antibodies for c-Myc (#631206), Flag (#635691) and HA(#631207) were from Clontech, and all the other antibodies, including Ac-lysine (AKL5C1, sc-32268), β-actin (sc-47778), eNOS (sc-654), ERβ (sc-137381), p300 (sc-585), PPARγ (sc-7196), p-Ser Antibody (4A3, sc-81516), RXR (sc-774), SIRT1 (sc-15404), and SOD2 (sc-30080) were obtained from Santa Cruz Biotechnology. 3-nitrotyrosine (3-NT) was measured by 3-Nitrotyrosine ELISA Kit (ab116691 from abcam) according to manufacturers’ instructions. The mitochondrial fraction was isolated using a Pierce Mitochondria Isolation Kit (Pierce Biotechnology) according to manufacturers’ instructions. Nuclear extracts were prepared using the NE-PER Nuclear and Cytoplasmic Extraction Reagents Kit (Pierce Biotechnology). Protein concentration was measured using the Coomassie Protein Assay Kit (Pierce Biotechnology). Plasmid DNA was transfected by LipofectamineTM reagent (Invitrogen). siRNA against p300, PPARγ, RXR and SIRT1 or non-specific siRNA (from Ambion) was transfected using Oligofectamine reagent (Invitrogen) according to manufacturers’ instructions.

**Isolation of mouse endothelial cells (MECs).** Isolation of endothelial cells from the heart (or aorta in some experiments) was performed following the previously described procedure(1). The isolated endothelial cells were further characterized by immunofluorescence staining with an antibody to the von Willebrandt factor (vWF). The MECs isolated from mice with the pLVX-Puro lentivirus injection treatment were further selected using 2µg/ml puromycin, and the P3 to P5 passages were used. Isolated MECs were maintained in DME medium with all of the endothelial cell supplements, plus charcoal-stripped Fetal Bovine Serum (#[12676029](http://www.lifetechnologies.com/order/catalog/product/12676029), Life Technologies) to remove traces of interfering basal estrogen. In some experiments, the MES were conditionally immortalized using a hTERT lentivirus vector with an extended life span to achieve higher transfection efficiency and experimental stability(2).

**Construction of plasmids and vectors.** The mouse genomic DNA was prepared from MECs. The ERβ gene promoter (2kb upstream of the transcription start site plus first exon) was amplified by PCR and subcloned into pGL3-basic vector using restriction sites of KpnI and HindIII with the following primers: Forward: 5’-gcgc-ggtacc-cat aaa tcc ttc cac ctc ctt -3’ (Kpn I) and Reverse: 5’-gcgc-aagctt-cta gcg ggt gga cat tct ctc-3’ (Hind III). To localize the SIRT1 responsive element from the ERβ promoter, the related deletion constructs were generated using PCR methods and the indicated mutation plasmids were generated using the Site-directed Mutagenesis Kit (Promega). All of the vectors were verified by sequencing, and more detailed information is available upon request. The cDNA for mouse SIRT1, PPARγ and p300 (obtained from Open Biosystems) were subcloned into pCMV-FLAG vector (#635688, ClonTech), pCMV-Myc and pCMV-HA vector (#631604, Clontech), respectively, to generate pCMV-FLAG-SIRT1, pCMV-Myc- PPARγ and pCMV-HA-p300 plasmids.

**RT reaction and real-time quantitative PCR.** Total RNA from treated cells was extracted using the RNeasy Mini Kit (for small amounts of cells isolated using laser capture microdissection techniques) or the RNeasy Micro Kit (Qiagen), and the RNA was reverse transcribed using an Omniscript RT kit (Qiagen). All of the primers were designed using Primer 3 Plus software with the Tm as 60°C, primer size as 21bp, and the product length in the range of 140-160bp (see Table S1). The primers were validated with the amplification efficiency in the range of 1.9-2.1, and the amplified products were confirmed with agarose gel. The real-time quantitative PCR was run on iCycler iQ (Bio-Rad) with the Quantitect SYBR green PCR kit (Qiagen). The PCR was performed by denaturing at 95°C for 8 min, followed by 45 cycles of denaturation at 95°C, annealing at 60°C, and extension at 72°C for 10s, respectively. 1 µl of each cDNA was used to measure target genes. The β-actin was used as the housekeeping gene for transcript normalization, and the mean values were used to calculate relative transcript levels with the ^ΔΔ^CT method according to instructions from Qiagen. Briefly, the amplified transcripts were quantified by the comparative threshold cycle method using β-actin as a normalizer. Fold changes in gene mRNA expression were calculated as 2^−ΔΔCT^ with CT = threshold cycle, ΔCT=CT(target gene)-CT(β-actin), and the ΔΔCT =ΔCT(experimental)-ΔCT (reference).

**Luciferase reporter assay.** 1.0×10^5^ cells were seeded in a 6-well plate with completed medium to grow until they reached 80% confluence. The related luciferase reporter plasmids (3µg) and 0.2µg pRL-CMV-Luc *Renilla* plasmid (from Promega) were transiently cotransfected, and in some experiments, the siRNA oligoneucleotides were cotransfected. After treatment, the cells were then harvested and the luciferase activity assays were carried out using the Dual-Luciferase^TM^ Assay System (Promega), and the transfection efficiencies were normalized using a cotransfected *Renilla* plasmid according to manufacturers’ instructions.

**Immunoprecipitation (IP).** Cell lysates or nuclear extracts were pre-cleared by pre-immune IgG plus Protein A agarose beads for 2 hr, and the supernatants were immunoprecipitated by the indicated antibodies and a 50% slurry of Protein A Agarose beads overnight at 4˚C. After washing with buffer containing 50 mM Tris, pH 7.5, 150 mM NaCl, 1% NP-40, and 0.5% deoxycholate with protease inhibitors, the precipitated proteins were measured by Western blotting.

**Western Blotting.** Cells were lysed in an ice-cold lysis buffer (0.137M NaCl, 2mM EDTA, 10% glycerol, 1% NP-40, 20mM Tris base, pH 8.0) with protease inhibitor cocktail (Sigma). The proteins were separated in 10% SDS-PAGE and further transferred to the PVDF membrane. The membrane was incubated with appropriate antibodies, washed and incubated with HRP-labeled secondary antibodies, and then the blots were visualized using the ECL+plus Western Blotting Detection System (Amersham). The blots were quantitated by IMAGEQUANT, and final results were normalized by β-actin.

**Chromatin Immunoprecipitation (ChIP).** Cells were washed and crosslinked using 1% formaldehyde for 20 min and terminated by 0.1M glycine. Cell lysates were sonicated and centrifuged. 500µg of protein were pre-cleared by BSA/salmon sperm DNA with preimmune IgG and a slurry of Protein A Agarose beads. Immunoprecipitations were performed with the indicated antibodies, BSA/salmon sperm DNA and a 50% slurry of Protein A agarose beads. Input and immunoprecipitates were washed and eluted, then incubated with 0.2mg/ml Proteinase K for 2h at 42˚C, followed by 6h at 65˚C to reverse the formaldehyde crosslinking. DNA fragments were recovered by phenol/chloroform extraction and ethanol precipitation. A 151bp fragment in the range of -400~-200 from the transcription start site on mouse ERβ promoter was amplified by real-time PCR (qPCR) using the below primers: forward 5’- tcg gtg cta tta ccc gaa ac-3’ and reverse 5’- cca ggg att ctg gac tta acc-3’.

**DNA Affinity Precipitation Assay (DAPA).** Biotin-labeled sense and antisense oligonucleotides for fragment (-330~-200) of mouse ERβ promoter were synthesized, annealed and purified as the DAPA probe. 200µg of nuclear extracts in binding buffer (60mM KCl, 12mM HEPES, pH7.9, 4mM Tris-HCl, pH 7.5, 5% glycerol, 0.5mM EDTA, 1mM DTT and protease inhibitors) were pre-cleared using 3µg of scrambled double-strand DNA supplemented with pre-equilibrated Tetralink™ Avidin Resin (Promega). The pre-cleared nuclear extracts were further incubated with 2µg of DAPA probe at 4˚C for 2h with gentle rotation, then 20µl of pre-equilibrated Tetralink™ Avidin Resin were added to incubate for another 1h. Beads were pelleted and washed with buffer, then boiled for 5min in SDS-PAGE gel loading buffer for western blotting analysis.

Measurement of ROS generation. Treated cells were seeded in a 96-well plate and incubated with 10μM CM-H2DCFDA (Invitrogen) for 45min at 37°C. Then, the intracellular formation of reactive oxygen species (ROS) was measured at excitation/emission wavelengths of 485/530nm using a FLx800 microplate fluorescence reader (Bio-Tek), and the data was normalized as arbitrary units(3).

Measurement of DNA breaks. Comet assay was measured using a CometAssay™ kit (Cat No. TA800) from R&D Systems Inc, and the 8-OHdG formation was measured using an OxiSelect™ Oxidative DNA Damage ELISA Kit (Cat No. STA320, from Cell Biolabs Inc.) according to manufacturers’ instructions. The formation of γH2AX was measured from nuclear extracts by western blotting using H2AX as input control.

**Evaluation of mitochondrial function.**

*Mitochondrial DNA copies.* The genomic DNA was extracted from treated MECs using a QIAamp DNA Mini Kit (Qiagen) and the mitochondrial DNA was extracted using the REPLI-g Mitochondrial DNA Kit (Qiagen). The purified DNA was used for the analysis of genomic β-actin (marker of the nuclear gene) and ATP6 (ATP synthase F0 subunit 6, marker of the mitochondrial gene) respectively using the qPCR method as mentioned above. The primers for genomic β-actin: forward 5’-acc aca gct gag agg gaa atc-3’ and reverse: 5’-cgt tgc caa tag tga tga cct -3’. The primers for ATP6: forward 5’-cgt aat tac agg ctt ccg aca-3’ and reverse 5’-ctg taa gcc gga ctg cta atg -3’. The mitochondrial DNA copies were obtained from relative ATP6 copies that were normalized by β-actin copies using the ^ΔΔ^CT method.

*Intracellular ATP level.* The intracellular ATP level was determined using the luciferin/ luciferase-induced bioluminescence system. An ATP standard curve was generated at concentrations of 10^-12^-10^-3^M. Intracellular ATP levels were calculated and expressed as nmol/mg protein(3).

*Caspase-3 activity.* The caspase-3 activity was determined using the ApoAlert caspase assay kit (Clontech). Treated cells were harvested and 50 µg of proteins were incubated with the fluorogenic peptide substrate Ac-DEVD-7-amino-4-trifluoromethyl coumarin (AFC). The initial rate of free AFC release was measured using a FLx800 microplate reader (Bio-Tek) at excitation/emission wavelengths of 380/505nm, and the enzyme activity was calculated as pmol/min/mg(3).

*Mitochondrial mass.* The mitochondrial mass was measured using FACS analysis after it was stained with Mitotracker Green FM (Molecular Probes,CA). Briefly, 1×10^6^ cells was stained for 45min with 100nM Mitotracker Green. Emission at 516nm was evaluated using a Becton Dickinson FACScan. Data was analyzed using FlowJo v7.5 (Tree Star Inc.), and the emission value was normalized from control.

*Mitochondrial membrane potential (Δψm).* The Δψm was measured by TMRE (from Molecular Probes T-669) staining. Prepare a 600μM T-669 stock solution using DMSO. Cells were grown on coverslips, and immerse the cells in 600nM TMRE for 20min at 37°C to load the cells with dye, then aspirate the labeling medium, and immerse the cells in 150nM TMRE to maintain the equilibrium distribution of the fluorophore. Mount the coverslips with live cells onto confocal microscope to image the cells using 548nm excitation/573nm emission filters. The intensity of TMRE fluorescence was measured using Image J software. Data from 10-20 cells were collected for each experimental condition and mean values of fluorescence intensity ± SEM were calculated.

*The OXPHOS proteins*. The OXPHOS proteins were measured using theTotal OXPHOS Rodent WB Antibody Cocktail (#ab110413, from Abcam) according to manufacturers’ instructions.

**The enzyme activity assay.**

*SIRT1 activity assay*. The SIRT1 deacetylase activity was evaluated in nuclear extract from MECs using a SIRT1 Fluorometric Drug Discovery Kit (Cat #: BML-AK555, Enzo Life Sciences) according to manufacturers’ instructions. Fluorescent intensity was measured using a FLx800 microplate fluorescence reader (Bio-Tek). No enzyme and Time 0 negative controls were generated by incubating developer II solution with 2mM nicotinamide before mixing the substrates with or without samples. SIRT1 activity was calculated with the corrected arbitrary fluorescence units of the tested samples to No-enzyme control and expressed as fluorescent units relative to the control(4).

*CK2 activity assay.* The Casein Kinase 2 activity was measured using a Casein Kinase 2 Assay Kit (#17-132 from Upstate) following manufacturers’ instructions.

*SOD2 activity assay*. The SOD2 was obtained from the mitochondrial fraction that was isolated using a Pierce Mitochondria Isolation Kit (Pierce) according to manufacturers’ instructions. The SOD activity was measured as described previously(5). Briefly, a stable O_2_^.-^ source was generated through the conversion action of XOD (xanthine oxidase) from xanthine and was mixed with chemiluminescent (CL) reagents to achieve a stable light emission. The SOD2 sample injection can scavenge O_2_^.-^ and the subsequent decrease of chemiluminescent response is proportional to the SOD2 activity. This system can have a detection limit of 0.001U.ml^-1^ with the linear range of 0.03~2.00U.ml^-1^. The results were normalized by protein concentration and were expressed as Units/mg proteins (U/mg).

*NOS activity assay.* The nitric oxide synthase (NOS) activity was measured by monitoring the conversion of [^3^H]-arginine to [^3^H]-citrulline using the NOS Activity Assay Kit (Cayman Chemical)(6). Briefly, the proteins from the endothelial cells were extracted and resuspended in a homogenization buffer (25mM Tris, pH 7.4/1mM EDTA/1mM EGTA) and centrifuged at 12,000rpm for 5min at 4°C. The reactions were performed with 25mM Tris (pH 7.4), 3μM BH_4_, 1μM FAD, 1μM FMN, 1mM NADPH (Sigma), 20 nCi/μl [^3^H]arginine, 600μM CaCl_2_, and 0.1μM calmodulin at room temperature for 30min. The assays were terminated by the addition of stop buffer (50 mM Hepes, pH 5.5/5 mM EDTA) followed by the addition of 100μl of equilibrated resin to remove the unconverted [^3^H]arginine. The elutes were transferred to a scintillation vial and the radioactivity was quantified by liquid scintillation spectrometry. The results were normalized by protein concentration.

**Generation of lentivirus**.

*Casein kinase 2 lentivirus*. The mouse cDNA for CK2 (casein kinase 2, from Open Biosystem) was subcloned into the pLVX-Puro vector (from Clontech) using the restriction sites of Xho1 and Xba1 with the below primers: Forward primer: 5’-gtac-ctcgag-atg tcg gga ccc gtg cca agc-3’ (Xho1) and Reverse primer: 5’-gtac-tctaga- tta ctg ctg agc gcc agc ggc-3’ (Xba1). Then, the CK2 was expressed through Lenti-X™ Lentiviral Expression Systems (from Clontech) according to manufacturers’ instructions. The virus was further purified, concentrated and titrated to reach ~2×10^8^ MOI per mL for the tail vein injection to infect the experimental mice.

*Tie2-driven ERβ/SIRT1 expression lentivirus.* The mouse genomic DNA was purified from C57BL/J6 wild type mouse, and the endothelium-specific Tie2 promoter (-1.2kb upstream) was amplified by PCR. The mouse cDNA for ERβ and SIRT1 was obtained from Open Biosystems. The cDNA for SIRT1 single mutant SIRT1-C152(D) was made from SIRT1 wild type (SIRT1-WT) using the Site-directed Mutagenesis Kit (Promega), and the SIRT1 amino acid at 152 was mutated from C (coded by TGT) to D (coded by GAT). The Tie2 promoter was fused with the mouse ERβ, SIRT1-WT or SIRT1-C152(D) cDNA, then subcloned into the pLVX-Puro vector (from Clontech) with the restriction sites of Xho1 and Xba1 using the below primers: ERβ forward primer: 5’-gtac-ctcgag-cag att gga agc att aca ggc-3’ (Xho1) and ERβ reverse primer: 5’-gtac-tctaga-agg ccc acg atg cta ggg tac-3’ (Xba1). SIRT1 forward primer: 5’-gtac-ctcgag- atg gcg gac gag gtg gcg ctc -3’ (Xho1) and SIRT1 reverse primer: 5’-gtac-tctaga- tta tga ttt gtc tga tgg ata -3’ (Xba1). The Tie2-empty, Tie2-ERβ or Tie2-SIRT1 was expressed through Lenti-X™ Lentiviral Expression Systems (from Clontech) according to manufacturers’ instructions. In order to evaluate the efficiency of SIRT1 lentivirus infection on the vascular wall, the primers specific for SIRT1-WT and SIRT1-C152(D) were designed as follows to distinguish the expression differences by qPCR. SIRT1-WT forward: 5’-gct ttc att cc TGT gaa agt ga -3’, SIRT1-WT reverse: 5’-ttt aag aat tgt tcg agg atc g-3’; SIRT1-C152(D) forward: 5’-gct ttc att cc GAT gaa agt ga -3’, SIRT1-C152(D) reverse: 5’-ttt aag aat tgt tcg agg atc g-3’.

*Tie2-driven ERβ shRNA lentivirus.* According to our preliminary data from *in vitro* cell culture experiments, the following sequence was confirmed as the most effective to knockdown mouse ERβ: 5’-ccg gag aac ggt gtg gtc atc aaa tct cga gat ttg atg acc aca ccg ttc ttt ttt tg-3’, and the shRNA template for ERβ or scrambled were designed (sense strand + loop + antisense strand) and the related double strand DNA (dsDNA) was synthesized and annealed. They were fused with mouse Tie2 promoter (-1.2kb upstream) by BamH1 sticky site at 5’-end and EcoR1 sticky site at 3’-end, and then inserted into pLVX-shRNA1 vector (from Clontech) using BamH1/EcoR1 restriction sites. The Tie2-scrambled (CTL) or Tie2-shERβ lentivirus was then expressed through Lenti-X™ shRNA Expression Systems (from Clontech) according to manufacturers’ instructions.

***In vivo* mouse experiments.** The animal protocol conformed to US NIH guidelines (Guide for the Care and Use of Laboratory Animals, No. 85-23, revised 1996), and was reviewed and approved by the Institutional Animal Care and Use Committee with approved document #:GDYAP2013001. The mice were housed 4 or 5 per cage on a 12:12-h light-dark cycle and were given phytoestrogen-free commercial rodent chow and water ad libitum on arrival.

To investigate the effect of estradiol on gene expression in the endothelium, the Young (6 months) and Old (30 months) mice were anaesthetized by intraperitoneal injection of 100mg/kg ketamine/16mg/kg xylazine, then given either sham, gonadectomy (GDX for male) or ovariectomy (OVX for female) surgery, and the mice received treatments consisting of 21-day time release pellets (Innovative Research of America, # E-121) that were implanted subcutaneously via a ~3-mm incision on the dorsal aspect of the neck. Hormone pellets contained 0.18mg of E2, while placebo pellets contained the same matrix as the E2 pellets but with no hormone (7). After 2 weeks of surgery with the treatment of either Vehicle (Veh) or Estradiol (E2), the mice were then sacrificed and the MECs were isolated from the thoracic aorta in treated mice using Laser Capture Microdissection (LCM) techniques to measure the mRNA expression by qPCR.

To investigate the effect of lentivirus carried gene expression on the vascular aging, the male mice (C57BL/6J) fed with high-fat diet (HFD, 60% calories from fat, Research Diets, #D12492) were used in this study throughout the experiments. The Young (4 months) and Old (28 months) mice received tail vein injections of 150µl of lentivirus (2×10^8^ MOI) for the Tie2-Empty (CTL), Tie2-↑ERβ, Tie2-↑SIRT1 or Tie2-shERβ twice within a 2-day interval. This injection procedure was administered again after one month. After 2 months, the Young (6ms) and Old (30ms) mice were overnight-fasted, euthanized by 100mg/kg pentobarbital, and the blood was collected for measuring plasma ^14^C-OA, estrogen (E2) and lipids, including total cholesterol, triglyceride, LDL and HDL cholesterol. The MECs from the heart were isolated for in vitro cell culture analysis, the MECs from the thoracic aortas (8) were picked up by Laser Capture Microdissection (LCM) for mRNA analysis. The carotid arteries were isolated to measure vessel tension. In some treatments, the hearts were dissected and snap-frozen in the OCT compound. The 10μm sections were cut using a clean microtome and mounted on PEN-membrane slides (2.0μm, Leica) for isolation of mouse endothelial cells (MECs) and mouse cardiomyocytes (MCMs) using Laser Capture Microdissection (LCM) for mRNA analysis. Also, in some experiments, the female OVX mice were used to briefly repeat the same experiments as we did in male mice. The details for these mice and their treatments are shown in Table S2.

**Isolation of bone marrow-derived endothelial progenitor cells (EPCs).** Primary bone marrow cells were collected from tibia in treated mice as indicated, and maintained in endothelial basal medium (EBM) with supplements of hydrocortisone, EGF, and 10% FCS on fibronectin/gelatin–coated dishes. The media was refreshed every day for 3 days, then the cells were stimulated with human recombinant VEGF for 2 days as described previously (9). The non-adherent cells were removed and collected every day as the control (CTL) cells. The adherent cells were characterized by washing with medium and incubating with 2.4ug/ml 1,1′-dioctadecyl-3,3,3′,3′-tetramethylindocarbocyanine-labeled acetylated LDL (Dil-Ac-LDL) for 1 hour. Cells were fixed in 2% paraformaldehyde and counterstained with FITC-labeled lectin. Double positive staining cells were considered to be endothelial progenitor cells (EPCs) (9). Both CTL and EPCs cells were used for mRNA analysis by qPCR.

**Evaluation of fatty acid metabolism**

*In vitro lipid transport assay*. Cells were seeded in a 12-well plate and grew until they were 80% confluent. After treatment, 0.5mCi well^-1^ of ^14^C-oleic acid (OA) from PerkinElmer was added. After 4h of incubation, the cells were washed and harvested, and the total radioactivity was quantitated by scintillation counting (10).

*Rate of fatty acid oxidation*. The fatty acid oxidation (FAO) rate was measured by evaluation of palmitate oxidation according to the published methods with minor modifications(11, 12). Briefly, the MECs from the heart and aorta in treated mice were cultured in T25 flask until they were 80% confluent, and the cells were starved for 2h in DMEM medium, then were incubated in DMEM containing 0.5% BSA/0.2mM palmitate/0.5μCi/mL 1-^14^C-palmitate at 37°C for 2h. The flasks were sealed at the beginning of the incubation with a stopper containing a filter (Whatman GF/B paper) pre-soaked in 5% NaOH. The incubation was stopped by the injection of 0.2 ml of 40% perchloric acid into each flask via a needle through the cap to acidify the medium and liberate the CO_2_. After overnight isotopic equilibration at room temperature, filters were removed, and the trapped ^14^CO_2_ and ^14^C acid-soluble products generated by the oxidation of [^14^C]palmitate were counted to calculate total palmitate oxidation. The protein concentrations were measured and the results were expressed as nmol per mg proteins per hour (nmol/mg/h).

*In vivo fatty acid uptake.* Mice were given a bolus dose of 2mCi of ^14^C-OA dissolved in olive oil through oral gavage. One hour after gavage, the blood sample was drawn from the tail vein for plasma analysis of radioactivity. Two hours after gavage, the mice were anaesthetized and perfused with PBS, and the heart, aorta and liver were dissected and dissolved overnight at 50µC in tissue solubilizer (1ml per 100mg tissue), then neutralized with 30µlml^-1^ glacial acetic acid. The total radioactivity was quantitated by scintillation counting(10).

*Plasma analysis for lipids*. The total cholesterol (TC), triglyceride (TG), LDL and HDL cholesterol in plasma was measured using a GM7 Micro-Stat Rapid Multiassay Analyser (Analox) according to manufacturers’ instructions.

**Monitoring of the vascular function**. The vessel tension for carotid artery in mice was measured in the Multi Wire Myograph System Model 620M (from Danish Myo Technology, Denmark). The three 3-mm aortic rings from each animal were quickly excised and placed in a Krebs bicarbonate buffer (118 mmol/L NaCl, 4.7mmol/L KCl, 25 mmol/L NaHCO3,1.2 mmol/L KH2PO4, 1.2 mmol/L MgSO4, 2.5 mmol/L CaCl2, and 5 mmol/L glucose), and the adhering tissue and fat were removed. Each ring was positioned between two 40-μm stainless steel wires in an 8-ml organ myograph chamber (DMT 620M), filled with Krebs bicarbonate buffer, maintained at 37±0.5°C and aerated with 95% O_2_ plus 5% CO_2_ (pH = 7.4). At the beginning of the experiment, each vessel ring was stretched to its optimal resting tension and allowed to equilibrate for 1h. To study vasodilator responses, the acetylcholine (Ach,10^−10^-10^–4^mol/l) induced vasodilation was assessed in aortas preconstricted with phenylephrine (10^–5^mol/l) at a level corresponding at least to the maximal response to potassium (100mmol/l KCl). The dose-response relaxation was measured for cumulative increments of acetylcholine at 1min intervals, and the Ach-induced change in tension was expressed as the percentage of the initial contraction induced by phenylephrine(6, 13).

**Radiotelemetric blood pressure monitoring in *vivo*.** Blood pressure was measured in conscious mice with the radiotelemetry technique described previously. Briefly, the mice were anaesthetized by intraperitoneal injection of 100mg/kg ketamine/16mg/kg xylazine, a catheter (PE10 tubing) and the telemetry transmitter unit (TA11PA-C10, Data Sciences International (DSI)) was implanted in the left carotid artery, while the radiotransmitter was placed in a subcutaneous pouch along the flank. Mice were treated with analgesics for 3 days (buprenorphine, 0.1 mg/kg) to relieve the pain and were allowed to recover for 7 days after surgery to regain their normal circadian rhythms before blood pressure measurements. While the blood pressure was being monitored, the mice were housed in a quiet room in individual cages placed above the telemetric receivers with an output to a computer. Blood pressure was measured for 5 minutes every hour, processed, and analyzed using the DataQuest ART system (DSI)(14-16).

**Statistical analysis**. The data was given as mean ± SEM, and all the experiments were performed at least in quadruplicate unless otherwise indicated. The one-way ANOVA and the Turkey−Kramer test was used to determine statistical significance of different groups by SPSS 18 software, a *P* value < 0.05 was considered significant.

REFERENCES

1. Takeshita K, Satoh M, Ii M, Silver M, Limbourg FP, Mukai Y, Rikitake Y, Radtke F, Gridley T, Losordo DW, et al. Critical role of endothelial Notch1 signaling in postnatal angiogenesis. *Circ Res.* 2007;100(1):70-8.

2. Bodnar AG, Ouellette M, Frolkis M, Holt SE, Chiu CP, Morin GB, Harley CB, Shay JW, Lichtsteiner S, and Wright WE. Extension of life-span by introduction of telomerase into normal human cells. *Science.* 1998;279(5349):349-52.

3. Yao D, Shi W, Gou Y, Zhou X, Yee Aw T, Zhou Y, and Liu Z. Fatty acid-mediated intracellular iron translocation: a synergistic mechanism of oxidative injury. *Free Radic Biol Med.* 2005;39(10):1385-98.

4. Hou X, Xu S, Maitland-Toolan KA, Sato K, Jiang B, Ido Y, Lan F, Walsh K, Wierzbicki M, Verbeuren TJ, et al. SIRT1 regulates hepatocyte lipid metabolism through activating AMP-activated protein kinase. *J Biol Chem.* 2008;283(29):20015-26.

5. Yao D, Vlessidis AG, Gou Y, Zhou X, Zhou Y, and Evmiridis NP. Chemiluminescence detection of superoxide anion release and superoxide dismutase activity: modulation effect of Pulsatilla chinensis. *Anal Bioanal Chem.* 2004;379(1):171-7.

6. Zhang W, Wang Q, Wu Y, Moriasi C, Liu Z, Dai X, Liu W, Yuan ZY, and Zou MH. Endothelial cell-specific liver kinase B1 deletion causes endothelial dysfunction and hypertension in mice in vivo. *Circulation.* 2014;129(13):1428-39.

7. Moran AL, Nelson SA, Landisch RM, Warren GL, and Lowe DA. Estradiol replacement reverses ovariectomy-induced muscle contractile and myosin dysfunction in mature female mice. *J Appl Physiol.* 2007;102(4):1387-93.

8. Wen L, Chen Z, Zhang F, Cui X, Sun W, Geary GG, Wang Y, Johnson DA, Zhu Y, Chien S, et al. Ca2+/calmodulin-dependent protein kinase kinase beta phosphorylation of Sirtuin 1 in endothelium is atheroprotective. *Proc Natl Acad Sci U S A.* 2013;110(26):E2420-7.

9. Dimmeler S, Aicher A, Vasa M, Mildner-Rihm C, Adler K, Tiemann M, Rütten H, Fichtlscherer S, Martin H, and Zeiher A. HMG-CoA reductase inhibitors (statins) increase endothelial progenitor cells via the PI 3-kinase/Akt pathway. *J Clin Invest.* 2001 Aug;108(3):391 - 7.

10. Hagberg CE, Falkevall A, Wang X, Larsson E, Huusko J, Nilsson I, van Meeteren LA, Samen E, Lu L, Vanwildemeersch M, et al. Vascular endothelial growth factor B controls endothelial fatty acid uptake. *Nature.* 2010;464(7290):917-21.

11. Taib B, Bouyakdan K, Hryhorczuk C, Rodaros D, Fulton S, and Alquier T. Glucose regulates hypothalamic long-chain fatty acid metabolism via AMP-activated kinase (AMPK) in neurons and astrocytes. *J Biol Chem.* 2013;288(52):37216-29.

12. Huynh FK, Green MF, Koves TR, and Hirschey MD. Measurement of fatty acid oxidation rates in animal tissues and cell lines. *Methods Enzymol.* 2014;542(391-405.

13. Rexhaj E, Paoloni-Giacobino A, Rimoldi SF, Fuster DG, Anderegg M, Somm E, Bouillet E, Allemann Y, Sartori C, and Scherrer U. Mice generated by in vitro fertilization exhibit vascular dysfunction and shortened life span. *J Clin Invest.* 2013;123(12):5052-60.

14. Cechova S, Zeng Q, Billaud M, Mutchler S, Rudy CK, Straub AC, Chi L, Chan FR, Hu J, Griffiths R, et al. Loss of collectrin, an angiotensin-converting enzyme 2 homolog, uncouples endothelial nitric oxide synthase and causes hypertension and vascular dysfunction. *Circulation.* 2013;128(16):1770-80.

15. Holobotovskyy V, Manzur M, Tare M, Burchell J, Bolitho E, Viola H, Hool LC, Arnolda LF, McKitrick DJ, and Ganss R. Regulator of G-protein signaling 5 controls blood pressure homeostasis and vessel wall remodeling. *Circ Res.* 2013;112(5):781-91.

16. Chamorro-Jorganes A, Grande MT, Herranz B, Jerkic M, Griera M, Gonzalez-Nunez M, Santos E, Rodriguez-Puyol D, Lopez-Novoa JM, and Rodriguez-Puyol M. Targeted genomic disruption of h-ras induces hypotension through a NO-cGMP-PKG pathway-dependent mechanism. *Hypertension.* 2010;56(3):484-9.

**Table S1. Sequences of primers for the real time**

**quantitative PCR (qPCR)**

| Gene | Species | Forward primer (5'→3') | Reverse primer (5'→3') |
| --- | --- | --- | --- |
| β-actin | Mouse | tcttgggtatggaatcctgtg | atctccttctgcatcctgtca |
| eNOS | Mouse | gcaggctctcacctacttcct | ctgaaccacttccattcttcg |
| ERα | Mouse | ccaaggagactcgctactgtg | aatggtgcattggtttgtagc |
| ERβ | Mouse | atgtgctatggccaacttctg | caagcttcctcttcagggtct |
| ERRα | Mouse | caggcttctcctcactgtcac | cccctcttcatctaggaccag |
| SIRT1 | Mouse | gtaagcggcttgagggtaatc | aaacttggactctggcatgtg |
| SOD2 | Mouse | ggcctacgtgaacaatctcaa | tcaggtttgtccagaaaatgg |

**Table S2. Details and conditions for the mice treatment**

| Animal Group | Young | Old | Old/  ↑ERβ | Young/  shERβ | Old/↑  SIRT1-WT | OVX/↑  SIRT1-C152(D) |
| --- | --- | --- | --- | --- | --- | --- |
| n | 13 | 14 | 12 | 11 | 13 | 15 |
| Age (months) | 6 | 30 | 30 | 6 | 30 | 30 |
| Lentivirus  Injection | Tie2-  Empty | Tie2-  Empty | Tie2-  ↑ERβ | Tie2-↑  shERβ | Tie2-  SIRT1-WT | Tie2-  SIRT1-C152(D) |

**Table S3.**

| Amino acid mutations for SIRT1 | MECs cells (Young/Old) | SIRT1 phosphorylation (p-Flag) verses  SIRT1-WT (%);  n; *P* value | ERβ mRNA expression verses SIRT1-WT (%);  n; *P* value |
| --- | --- | --- | --- |
| S154A | Young | 67±6; n=3; *P*<0.05 | 77±8; n=3; *P*<0.05 |
| S154A | Old | 76±7; n=3; *P*<0.05 | 82±5; n=3; *P*<0.05 |
| S154D | Old | 79±10; n=3; *P*<0.05 | 77±8; n=3; *P*<0.05 |
| S154E | Old | 84±8; n=3; *P*<0.05 | 75±7; n=3; *P*<0.05 |
| S154Q | Old | 76±11; n=4; *P*<0.05 | 83±6; n=4; *P*<0.05 |
| D155R | Old | 78±7; n=3; *P*<0.05 | 76±8; n=3; *P*<0.05 |
| E153R | Old | 68±9; n=3; *P*<0.05 | 78±10; n=3; *P*<0.05 |
| D156R | Old | 81±8; n=3; *P˂*0.05 | 74±9; n=3; *P*<0.05 |
| C152R | Old | 71±11; n=3; *P*<0.05 | 75±8; n=3; *P*<0.05 |
| C152E | Old | 127±10; n=4; *P*<0.05 | 121±9; n=4; *P*<0.05 |
| C152D | Old | 139±6; n=4; *P*<0.05 | 148±11; n=4; *P*<0.05 |

**Table S3. Effects of C152, E153, S154, D155 and D156 mutations on the SIRT1 phosphorylation and ERβ expression in MECs cells.** The conditionally immortalized MECs from either Young or Old mice were transfected by either Flag-SIRT1 WT (wild type) or Flag-SIRT1 single mutants for further analysis. The SIRT1 phosphorylation was measured by IP/WB of Flag-SIRT1/p-Flag, and the ERβ mRNA was measured by qPCR. n is the number of independent test. The *P* value was measured by ANOVA test, and the *P* <0.05 means significant difference vs SIRT1-WT group. Results are expressed as mean ± SEM.

**Figure S1**

**Figure S1**. **Decreased ERβ expression is due to compromised phosphorylation at aa S154 in SIRT1, the mutant SIRT1-C152(D) restores this effect in the endothelium from aging mice.** (a-d). The MECs from either Young or Old mice were infected with either control or CK2 lentivirus, and the cells were then harvested for further analysis. (a) CK2 activity. (b) Quantitation for Fig 3c. (c) Quantitation for Fig 3d. (d) Quantitation for 3e. n=4. *, *P*<0.05, vs Young group. (e-g) The conditional immortalized MECs from Young mice were transfected by either Flag-SIRT1 WT (wild type) or SIRT1 single mutants for further analysis. (e) Quantitation for Fig 3h. (f) Quantitation for Fig 3i. (g) Quantitation for Fig 3j. n=4. *, *P*<0.05, vs WT group; ¶, *P*<0.05, vs S154(Q) group. (h) Quantitation for Fig 3k. n=4, *, *P*<0.05, vs Young/WT group. (i-k) The MECs from either Young or Old mice were infected by either SIRT1-WT or single mutant SIRT1-C152(D) lentivirus, and the cells were harvested for further analysis. (l) Quantitation for Fig 3n. (j) Quantitation for Fig 3o. (k) Quantitation for Fig 3p. n=4. *, *P*<0.05, vs Young/WT group; ¶, *P*<0.05, vs Old/WT group. Results are expressed as mean ± SEM.

**Figure S2**

**Figure S2**. **Tie2-driven lentivirus expression is specific in endothelium, instead of other cells.** The mouse endothelial cells (MECs) and mouse cardiomyocytes (MCMs) were isolated from the hearts with indicated treatments using Laser Capture Microdissection (LCM) techniques, and the mRNA level was measured by qPCR. (a) ERβ mRNA, n=4. (b) SIRT1 mRNA, n=4. *, *P*<0.05, vs Young group. Results are expressed as mean ± SEM.

**Figure S3**

**Figure S3**. **Tie2-driven lentivirus expression of SIRT1-WT and SIRT1-C152(D) is specific in endothelium.** The MECs were isolated from the hearts with indicated treatments using Laser Capture Microdissection (LCM) techniques, and the mRNA levels, specific for the SIRT1 wild type (SIRT1-WT) and the SIRT1 single mutant C152(D) (SIRT1-C152(D)), were measured by qPCR. The following primers were used to distinguish the expression differences of SIRT1-WT and SIRT1-C152(D). SIRT1-WT forward: 5’-gct ttc att cc TGT gaa agt ga -3’, SIRT1-WT reverse: 5’-ttt aag aat tgt tcg agg atc g-3’; SIRT1-C152(D) forward: 5’-gct ttc att cc GAT gaa agt ga -3’, SIRT1-C152(D) reverse: 5’-ttt aag aat tgt tcg agg atc g-3’. (a) SIRT1-WT mRNA, n=4. (b) SIRT1-C152(D) mRNA, n=4. *, *P*<0.05, vs Young group. Results are expressed as mean ± SEM.

**Figure S4**

**Figure S4**. **Tie2-driven lentivirus expression is specific in endothelium, instead of other tissues.** Different tissues, including liver, kidney and hypothalamus were isolated from treated mice, and the mRNA level was measured by qPCR. (a) ERβ mRNA, n=4. (b) SIRT1 mRNA, n=4. *, *P*<0.05, vs Young group. Results are expressed as mean ± SEM.

**Figure S5**

**Figure S5**. **Tie2-driven lentivirus infection through tail vein injection has no significant effect on myeloid EPCs.** The endothelial progenitor cells (EPCs) and the related control (CTL) cells were isolated from bone marrow in treated mice and characterized for the analysis of mRNA level by qPCR. (a) ERβ mRNA, n=4. (b) SIRT1 mRNA, n=4. Results are expressed as mean ± SEM.

**Figure S6**

**Figure S6. Expression of ERβ and the mutant SIRT1-C152(D) restores mitochondrial dysfunction in the endothelium from aging mice.** The MECs were isolated from the hearts with indicated treatments for further analysis. (a) Mitochondrial DNA copies, n=4. (b) Caspase-3 activity, n=5. (c) Intracellular ATP levels, n=4. (d) Mitochondrial mass, n=5. (e) Quantitation of Mitochondrial membrane potential (Δψm) level, n=4. (f) Quantitation of OXPHOS proteins, n=5. (g) Representative western blotting band for (g). *, *P*<0.05, vs Young group; ¶, *P*<0.05, vs Old group; #, *P*<0.05, vs Old/↑SIRT1-WT group. Results are expressed as mean ± SEM.

.

**Figure S7**

**Figure S7. Expression of ERβ and the mutant SIRT1-C152(D) restores dysfunction of fatty acid metabolisms in the endothelium from aging mice.** (a,b) The MECs were isolated from the hearts with indicated treatments for further analysis. (a) The in vitro ^14^C-OA fatty acid uptake, n=4. (b) The in vitro palmitate oxidation rate, n=4. (c-e) The mice were infected by the indicated lentivirus through tail vein injection. The treated mice were given a bolus dose of 2mCi of ^14^C-OA through oral gavage, and the blood and tissues, including the heart, aorta and liver, were dissected for analysis of total radioactivity. (c) The in vivo ^14^C-OA uptake from the heart and aorta in 2h, n=9; (d) The in vivo ^14^C-OA uptake in plasma in 1h, n=7. (e) The in vivo ^14^C-OA uptake from liver in 2h, n=8. *, *P*<0.05, vs Young group; ¶, *P*<0.05, vs Old group; #, *P*<0.05, vs Old/↑SIRT1-WT group. Results are expressed as mean ± SEM.

**Figure S8**

**Figure S8. Expression of ERβ and the mutant SIRT1-C152(D) on the vascular wall ameliorates the vascular damage in aging female mice.** (a) The MECs were isolated from the heart in treated female OVX mice using Laser Capture Microdissection techniques to measure ERβ mRNA level by qPCR. n=4. (b) The treated female OVX mice were used to measure the mean of systolic blood pressure, n=10. *, *P*<0.05, vs Young group; ¶, *P*<0.05, vs Old group; #, *P*<0.05, vs Old/↑SIRT1-WT group. Results are expressed as mean ± SEM.
